# Cannabis policy priorities and public health: a survey of elected officials in California

**DOI:** 10.1186/s12889-026-27889-x

**Published:** 2026-05-29

**Authors:** Ryan Whitacre, Alisa Padon, Bethany Simard, Lynn D. Silver

**Affiliations:** 1 Prevention Policy Group, Public Health Institute, Berkeley, CA USA; 2 Division of Prevention Science, School of Medicine, University of California, San Francisco, CA USA; 3 Department of Epidemiology and Biostatistics, University of California, San Francisco, CA USA

**Keywords:** Cannabis policy, Public health, Local government, Elected officials, Partisan politics, Youth prevention

## Abstract

**Background:**

Cannabis legalization has substantial impacts on public health across the United States, yet the perspectives of local elected officials who oversee regulatory environments and make decisions about cannabis business retail licensing, taxation, and marketing restrictions remain largely unstudied. Understanding how these policymakers prioritize health considerations relative to economic and other concerns is essential for developing effective public health advocacy and anticipating barriers to implementing health-protective cannabis regulations.

**Methods:**

From September 2023 to February 2024, we conducted an online survey of 2,681 elected officials in California local government, assessing cannabis policy positions, priorities, and engagement. Descriptive statistics were calculated for all measures. Likelihood ratio tests using logistic regression assessed differences across political party affiliations (Democrat, Republican, Independent), with analyses stratified by whether respondents’ jurisdictions allowed cannabis retail. One open-ended measure was analyzed using thematic coding.

**Results:**

Among 250 respondents (9.3% response rate), who were primarily city council members, 40% reported direct cannabis policy experience and 75% expressed interest in regulatory involvement. Top priorities were tax revenue (41%), economic development (41%), and youth cannabis use (38%), reflecting tension between fiscal and health considerations. Support for licensing adult-use retailers was significantly higher among Democrats (72%) than Republicans (39%; OR = 0.25 [95% CI: 0.11, 0.56]) or Independents (38%; OR = 0.23 [0.10, 0.53]). Republicans were significantly more likely to prioritize adverse health effects (47% vs. 22% Democrats; OR = 3.10 [1.36, 7.07]). Social equity was prioritized by only 13% overall, with significant partisan variation (20% Democrats vs. 3% Republicans; OR = 0.11 [0.01, 0.85]). Cross-party agreement emerged on restricting youth-attractive packaging (84%), yet fewer than 4% of California jurisdictions that allow retail to operate have implemented such protections. Qualitative responses revealed diverse framings of cannabis—from prohibition-era moral concerns to wellness narratives—suggesting policy decisions are often not evidence-based.

**Conclusions:**

This first systematic study of U.S. elected officials’ cannabis policy positions documents a political landscape in which economic considerations predominate over health concerns and significant gaps exist between policymakers’ support for health-protective measures and actual policy implementation. These findings identify actionable opportunities for evidence-based public health advocacy, particularly in support of bipartisan youth protection measures.

**Supplementary Information:**

The online version contains supplementary material available at 10.1186/s12889-026-27889-x.

## Background

Cannabis legalization represents one of the most significant natural policy experiments affecting public health in the United States [1]. While cannabis remains federally illegal in the U.S. as a controlled substance, thirty-one individual states (and Washington, DC) have decriminalized cannabis use, and twenty-four have legalized cannabis for adult use [2]. At the same time, scientific evidence has associated cannabis use with increased risk of cannabis use disorder, adverse mental health and perinatal outcomes, and cardiovascular disease [1, 3–7]. Further evidence suggests that the public health impact of state legalization is not uniform – it is shaped by local control and regulatory decisions that vary significantly across jurisdictions [8–12]. Understanding how local elected officials prioritize and frame cannabis policy issues is therefore essential for predicting and influencing public health outcomes.

Health equity considerations are central to cannabis policy. cannabis retailers may disproportionately concentrate in lower-income and minority communities [9], potentially exacerbating existing health disparities. Meanwhile, the legacy of racialized enforcement of cannabis prohibition has produced profound health and socioeconomic harms in communities of color [13]. Local policy decisions about retail licensing, taxation, and social equity programs may determine whether legalization ameliorates or exacerbates these disparities. Yet we know little about how elected officials who make these decisions prioritize health equity relative to other considerations.

The liberalization of cannabis policy has also introduced a complex set of laws and policies aiming to eliminate the illegal market, safeguard and regulate the legal market, and provide access to medicinal and/or adult-use cannabis. Twenty-six states and D.C. offer some form of expungement of criminal records for previous cannabis offenses [14, 15], and several have established licensing programs for groups disproportionately impacted by the War on Drugs [16]. In this increasingly favorable policy environment, the legal cannabis industry has grown rapidly, intensifying marketing practices and competition among cannabis businesses [17]. As a result, these businesses now produce a wide range of product types at increasingly higher potency levels [18], collectively generating significant revenue. In 2025, annual sales in the US market are estimated to reach $34 billion [19].

Policymakers in states throughout the country are thus responsible for decision-making on whether and how to legalize cannabis businesses, often addressing complex cannabis policy issues, which may have significant public health impacts. Elected officials in city and county government play an essential role in decision-making in cannabis policy because most states with legal markets have at least some degree of ‘local control’ in their approach to regulating commercial cannabis [20]. Local control in California is robust, allowing local jurisdictions to take policy actions, including permitting or prohibiting retail adult-use sales, restricting the products allowed to be sold, limiting marketing that is attractive to youth, and adopting local taxes [21, 22].

Evidence suggests that local cannabis policies impact public health outcomes, and unequal policy implementation could exacerbate existing social and health inequities [9, 10, 23]. For example, higher proximity to storefront retailers and retail outlet density near homes has been associated with increased adolescent cannabis use and problematic use, as well as use during pregnancy in Northern California [24, 25]. Local retail bans and longer drive times to the nearest retailer were associated with a markedly lower prevalence of psychotic disorder diagnoses in California adolescents, and greater retailer density with higher rates of anxiety and depressive disorder diagnoses [12]. Frequent use by California adolescents increased post-legalization and is higher in schools in communities that allowed storefront retail sales [11]. The significant increase in cannabis use during pregnancy in California post-legalization was limited to local jurisdictions that allowed recreational retailers [6]. Packaging, marketing, and product-type restrictions drawn from tobacco control have been linked to lower appeal and availability of youth-attractive products and vary locally [26–28]. It is thus vital to understand how elected officials in state, county, and city governments engage with cannabis policy.

This study adopts a translational approach to public health research, recognizing that understanding policymakers’ perspectives is essential for developing effective public health advocacy and informing the translation of evidence into policy [29]. By examining how elected officials frame and prioritize cannabis policy issues, we can identify opportunities for evidence-based intervention and anticipate barriers to implementing robust public health protections, such as regulatory measures with demonstrated effectiveness in analogous commercial markets: restrictions on youth-attractive packaging and product types; outlet density caps and school/residential buffer zones; marketing and signage restrictions; dedicated cannabis tax revenue for prevention and treatment; and child-resistant packaging and potency labeling [1]. This study addresses three research questions with direct implications for public health: (1) How do elected officials prioritize cannabis policy issues, and to what extent do health considerations feature in their priorities? (2) How are partisan affiliations associated with policy positions that may differentially affect public health outcomes? (3) What gaps exist between policymakers’ perceptions of effective youth prevention strategies and current regulatory practice?

This study also advances scholarship in critical drug policy studies, which have illustrated that drug policy is a contested terrain overdetermined by politics, morality, and governance, not just evidence [30, 31]. We know from significant ethnographic research that state policies criminalize informal markets while regulating and profiting from formal drug markets [32, 33]. Political ideologies and other sociocultural forces shape how drug-related harms, responsibilities, and solutions are framed [34]. Since US drug policy has been deeply rooted in carceral logics and processes of marginalization, the language of “disorder” and “urban decay” in policy discourse served to obscure structural inequalities and reinforce marginalization [13, 34]. Scholars have shown how the rhetorical construction of ‘drug epidemics’ can fuel moral panics, with drugs used symbolically to represent broader social anxieties about disorder, deviance, and the breakdown of the social fabric [35]. Indeed, a critical lens is essential to a more comprehensive understanding of elected officials’ cannabis policy positions and priorities.

Yet, no previous research in the United States has systematically assessed elected officials’ cannabis policy priorities and positions. However, some related work has been published recently in the US and elsewhere. A study assessing the sentiment of US policymakers in cannabis policy discourse on Twitter found that politicians primarily authored informative tweets with neutral tones [36]. Another study in Ohio found that cannabis legalization has encountered legislative and public resistance [37]. In Canada, researchers have examined how federal legalization interacted with provincial and municipal regulatory choices, shaping retail access and use patterns, and the complex policymaking process around Quebec’s innovative public monopoly approach [38, 39]. An Irish study exploring policymakers’ attitudes toward the decriminalization and legal regulation of adult-use cannabis found broad support for decriminalization but qualified support for the legal regulation of the cannabis industry [40]. The Irish study also found that policymakers were willing to offer opinions that contrasted with their organizations’ policy positions [40]. In Denmark, researchers examining the rhetorical framing of cannabis use in policy discourse found that representations of cannabis as a singular object are being replaced by perspectives that highlight the multiple ontologies of cannabis use; as, respectively, a social problem, a problem of deviance, an organized crime problem, a health- and risk problem, and as a medical problem [41].

This is the first study to assess the cannabis policy positions and priorities of elected officials in the US, examine the role of political party affiliation, and pinpoint priority cannabis policy issues, including public health topics. It also uniquely assesses how elected officials frame their policy positions and priorities, drawing insights from critical scholarship on drug policy. The study was conducted in California, a crucial site for this research, as it is the largest cannabis market in the world [42] and allows for substantial local control [20], offering methodological and empirical insights applicable to many other local and state governments.

## Methods

### Study design and sample

We conducted an online survey of California elected officials (or their staffers responding on their behalf) in city, county, and state government. The sample comprised all City Council Members, County Supervisors, County Sheriffs, District Attorneys, and Members of the State Legislature (or their staff) with valid emails listed on jurisdiction websites, yielding 2,681 invited participants. Invitations were sent on September 23, 2023, with reminders during study weeks 2, 5, 9, and 12; the survey closed on February 2, 2024. Participant jurisdiction was validated by comparing self-reported responses with each respondent’s email domain (e.g., @city.ca.gov), deferring to email domain when discrepancies arose (*n* = 11), and using the elected official’s webpage when jurisdiction could not otherwise be ascertained (*n* = 7). State senators and assembly members were not assigned a jurisdiction affiliation. The study was reviewed and approved by the Institutional Review Board of the Public Health Institute (protocol I22-008) and adhered to the Declaration of Helsinki; all respondents provided informed consent.

### Survey instrument

The instrument was developed specifically for this study. Item development drew on (1) the cannabis policy and tobacco control literatures, which identified evidence-based environmental prevention strategies (retail density limits, marketing and packaging restrictions, local taxation, product safety); [22, 43] (2) prior scoping conversations with California elected officials and public health practitioners, which helped identify the policy issues and barriers most salient to local decision-makers; and (3) prior surveys of local government officials, which informed item formats and the use of forced-choice “top three” priority items [44]. Draft items were reviewed by the research team for face validity, clarity, neutrality of wording, and appropriateness to officials’ time constraints. Items covered four domains: (a) prior experience with, and barriers to, cannabis policy engagement; (b) substantive priorities (respondents’ own priorities and their perception of constituent priorities); (c) positions on specific regulatory measures (retail licensing, youth-attractive packaging and products, marketing, delivery, local taxation, and tax revenue allocation); and (d) stakeholder input received. The survey collected 18 closed-ended measures plus one open-ended item inviting additional perspectives (see. *Qualitative Analysis* below). Four measures used a forced-choice “top three” format: the cannabis policy issues most important to (1) the respondent and (2) their constituents, (3) priority uses of cannabis tax revenue, and (4) regulatory actions most effective for reducing youth cannabis use. The full survey instrument is provided as Additional File 1.

### Statistical analyses

Descriptive statistics were calculated for each measure, with percentages based on non-missing responses. We used likelihood ratio tests from logistic regression to compare responses across political party affiliations: Democrat, Republican, and Independent (those responding “Independent,” “no affiliation,” or “no party preference”) [45]. In cases of significance, each pair of parties was compared using Z-tests with a Bonferroni correction. Firth logistic regression, a penalized likelihood method, and Wald tests were used when quasi-complete separation was detected [46]. All analyses were repeated stratified by whether the respondent’s jurisdiction allowed cannabis retail as of January 1, 2024, using the Public Health Institute’s California Cannabis Policies database [47].

### Qualitative analysis

The single open-ended item was included by design. The instrument was structured to generate quantitative, breadth-oriented data across a large sample of elected officials whose availability for lengthy instruments is highly constrained [44], with one opportunity for rationales and framings not captured by closed-ended items to surface. Greater qualitative depth was pursued through a companion in-depth interview study with 20 California local elected officials, reported separately.

Sixty-two respondents (25%) provided open-ended text, ranging from brief phrases to multi-sentence paragraphs. We analyzed these responses using reflexive thematic analysis (Braun and Clarke) with a hybrid deductive-inductive coding strategy [48]. Initial codes were oriented deductively around the survey’s conceptual domains (policy priorities, positions on legalization and commercial regulation, stakeholder dynamics, health concerns, tax revenue, and implementation challenges); additional codes emerged inductively, proving especially important for capturing moral and symbolic framings of cannabis (e.g., prohibition-era concerns, wellness narratives, and consumer-protection framings). Coding was conducted in Microsoft Excel, with each segment assigned one or more codes. The lead author (RW) developed an initial codebook and iteratively refined it through team discussion; a second team member independently coded a subset of responses, with discrepancies resolved through consensus. Themes were synthesized by reading across coded segments, triangulating with quantitative results, and selecting illustrative quotations representing the range of positions. Syntheses were reviewed by all authors, with reflexive attention to how our public health and cannabis policy expertise shaped interpretation.

## Results

250 responses were received: 235 from elected officials and 15 from staffers responding on behalf of elected officials (Table 1), yielding a response rate of 9.3% (250/2,681). 80% of respondents were City Councilmembers or Mayors (*n* = 202). Other respondents were County Supervisors (11%), County Sheriffs (3%), District Attorneys (1%), and State Legislators (4%). 36% of respondents (*n* = 89) did not report their political party affiliation. Of those who did (*n* = 161), 53% identified as Democrats, 24% Republicans, and 23% Independents. Among local elected officials representing a single jurisdiction, 53% served jurisdictions with legalized retail cannabis sales as of January 1, 2024.

**Table 1:** Descriptive table of respondents and respondents’ jurisdiction characteristics

Position	Total (*n* = 250)	Political Party Affiliation				Local cannabis retail policy		Included in Political Affiliation Analysis (*n* = 155)	Provided open-ended response (*n* = 62)
		Democrat (*n* = 85)	Republican (*n* = 39)	Independent (*n* = 37)	No response (*n* = 89)	Allows (*n* = 127)	Bans (*n* = 112)		
	*n* (col %)	*n* (row %)				*n* (row %)		*n* (row %)	*n* (row %)
City Council	202 (81%)	77 (38%)	30 (15%)	31 (15%)	64 (32%	113 (56%)	89 (44%)	137 (68%)	49 (24%)
Member	200 (80%)								
Staffer	2 (0.1%)								
Board of Supervisors	27 (11%)	4 (15%)	7 (26%)	4 (15%)	12 (44%)	10 (37%)	17 (63%)	14 (52%)	
Member	24 (10%)								10 (37%)
Staffer	3 (1%)								
County District Attorney	2 (1%)	0 (0%)	0 (0%)	1 (50%)	1 (50%)	0 (0%)	2 (100%)	1 (50%)	0 (0%)
County Sheriff	8 (3%)	0 (0%)	1 (13%)	1 (13%)	6 (75%)	4 (50%)	4 (50%)	2 (25%)	0 (0%)
State Senate or Assembly	11 (4%)	4 (36%)	1 (9%)	0 (0%)	6 (55%)	NA	NA	0 (0%)	0 (0%)
Member	1 (0.4%)								
Staffer	10 (4%)								

Political affiliation analytic sample includes participants who identified as Democratic, Republican, or Independent, represented a single jurisdiction (i.e., no state position), and answered all questions

Most respondents (72%) completed all the multiple-choice measures of interest. Sixty-two responded to the open-ended questions on cannabis policy priorities. The sample for the logistic regression analyses excluded those who did not answer the political affiliation question (*n* = 89), represented more than one jurisdiction (i.e., state senators and assemblymembers) (*n* = 5), and did not answer all questions of interest (*n* = 1), yielding a sample size of 155 (80 Democrats, 38 Republicans, 37 Independents), 73 of which lived in a jurisdiction that banned retail, and 82 in jurisdictions allowing retail.

**Fig. 1:**
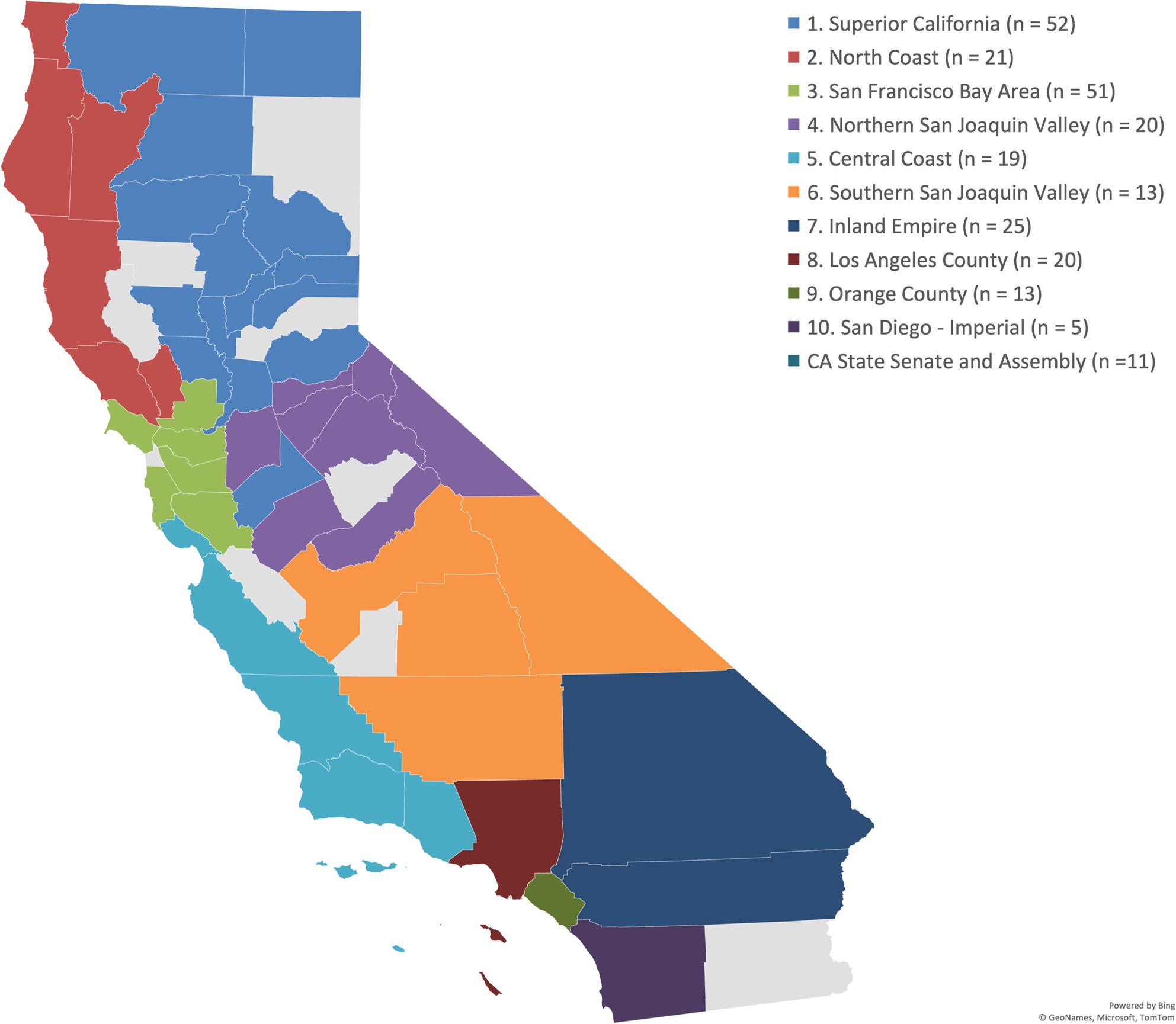
Geographic representation of respondents (elected officials) by county and Census region

Figure 1 illustrates the geographic distribution of respondents, who represented jurisdictions across all 10 US Census regions in California. The greatest number of responses was received from elected officials representing jurisdictions in Superior California (*n* = 52) and the San Francisco Bay Area (*n* = 51).

Forty percent of respondents (*n* = 99) reported direct involvement in cannabis policy in California (Table 2). Across specific policy issues, experience with cannabis taxation was reported most often (21%), followed by establishing legal commerce (20%) and implementing rules for business operations (20%). Experience was least common for marketing (3%), cannabis use prevention or treatment (6%), and opposition to legal commerce (7%) – i.e., efforts to prohibit cannabis retail in the respondent’s jurisdiction. Experience in these issues did not vary significantly by political party affiliation overall or stratified by local cannabis retail policy.

**Table 2:** Cannabis policy positions and priorities, total sample and by political affiliation

	Total	Political Affiliation Analysis Sample^a^		
		Democrats *n* = 80	Republicans *n* = 38	Independents *n* = 37
	*n* (col %)	*n* (col %)	*n* (col %)	*n* (col %)
Experience in cannabis policy (*n* = 245)				
Ever had direct involvement in cannabis policy in California^b^	99 (40%)	32 (40%)	14 (37%)	16 (43%)
Policy issue experience^c^ (*n* = 238)				
Taxation	51 (21%)	26 (32%)	6 (16%)	6 (16%)
Implementing rules for business operations	47 (20%)	22 (28%)	7 (18%)	9 (24%)
Establishment of legal commerce	47 (20%)	20 (25%)	7 (18%)	11 (30%)
Use of tax revenue	33 (14%)	13 (16%)	5 (13%)	9 (24%)
Eliminating the illicit market	27 (11%)	8 (10%)	4 (11%)	5 (14%)
Public education	25 (11%)	11 (14%)	4 (11%)	7 (19%)
Equity in licensing	19 (8%)	10 (12%)	0 (0%)	5 (14%)
Opposition to legal commerce	16 (7%)	2 (2%)	5 (13%)	1 (3%)
Cannabis use prevention or treatment	14 (6%)	7 (9%)	0 (0%)	4 (11%)
Marketing	6 (3%)	3 (4%)	0 (0%)	0 (0%)
Interest in cannabis industry regulation^d^ (*n* = 185)				
Somewhat or very interested in being involved	138 (75%)	62 (78%)	29 (76%)	25 (68%)
Cannabis policy is priority	110 (59%)	53 (66%)	21 (55%)	18 (49%)
Support for cannabis legalization in local jurisdictions^e^ (*n* = 191)				
Licensing of medical retailers	107 (56%)	60 (75%)	16 (42%)	13 (35%)
Licensing of adult-use retailers	101 (53%)	58 (72%)	15 (39%)	14 (38%)
Licensing other non-retailer business types	97 (51%)	49 (61%)	16 (42%)	17 (46%)
Allowing outside jurisdiction delivery	79 (41%)	44 (55%)	10 (26%)	13 (35%)
Approached by stakeholders (% yes)^d^ (*n* = 185)				
Private citizens	101 (56%)	43 (54%)	21 (55%)	20 (54%)
Cannabis industry representatives	97 (54%)	44 (55%)	21 (55%)	20 (54%)
Most important cannabis policy issues among elected officials^b^ (*n* = 191)				
Tax revenue	79 (41%)	40 (50%)	16 (42%)	14 (38%)
Economic development	78 (41%)	37 (46%)	13 (34%)	13 (35%)
Youth cannabis use	73 (38%)	26 (32%)	16 (42%)	18 (49%)
Eliminating the illicit market	65 (34%)	24 (30%)	15 (39%)	12 (32%)
Adverse health or social effects of cannabis use	57 (30%)	18 (22%)	18 (47%)	10 (27%)
Environmental issues	27 (14%)	9 (11%)	5 (13%)	4 (11%)
Social equity	24 (13%)	16 (20%)	1 (3%)	2 (5%)
Criminal justice	22 (12%)	13 (16%)	2 (5%)	4 (11%)
Most important cannabis policy issues among constituents^c^ (*n* = 191)				
Negative health impacts of youth use	81 (42%)	37 (46%)	18 (47%)	11 (30%)
Eliminating the illicit market	68 (36%)	29 (36%)	10 (26%)	13 (35%)
Negative community impacts of cannabis business	66 (35%)	23 (29%)	18 (47%)	14 (38%)
Negative health impacts of cannabis consumption	32 (17%)	9 (11%)	13 (34%)	3 (8%)
Negative social impacts of cannabis use, such as on work or education	23 (12%)	7 (9%)	9 (24%)	4 (11%)
Most effective regulatory actions for youth prevention^d^ (*n* = 191)				
Not allowing packaging attractive to youth	160 (84%)	72 (90%)	33 (87%)	27 (73%)
Limiting products mimicking candies or foods attractive to youth	105 (55%)	49 (61%)	21 (55%)	18 (49%)
Eliminating the illicit market	101 (53%)	44 (55%)	20 (53%)	20 (54%)
Limiting/prohibiting flavored inhaled products	76 (40%)	38 (48%)	12 (32%)	13 (35%)
Using tax revenue for youth programs	36 (19%)	22 (28%)	3 (8%)	7 (19%)
Education campaigns	31 (16%)	13 (16%)	4 (11%)	7 (19%)
Establishing larger buffer zones from retailers	30 (16%)	16 (20%)	2 (5%)	5 (14%)
Priority uses of cannabis tax revenue (*n* = 191)				
General fund with spending flexibility	105 (55%)	50 (62%)	19 (50%)	18 (49%)
Law enforcement	77 (40%)	22 (28%)	27 (71%)	16 (43%)
Community reinvestment	68 (36%)	31 (39%)	11 (29%)	9 (24%)
Public education on cannabis or substance use disorder	59 (31%)	26 (32%)	10 (26%)	14 (38%)
Preventing substance use disorder	43 (23%)	20 (25%)	7 (18%)	10 (27%)
Supporting vulnerable youth	42 (22%)	23 (29%)	4 (11%)	7 (19%)

Percentages are based on non-missing responses. Denominators vary due to item nonresponse

^a^Respondents who identified as Democrat, Republican, or Independent/have no affiliation, excluding those who did not provide a political affiliation (*n* = 89), represented more than one jurisdiction (i.e., state senators or assemblymembers, *n* = 11) and who did not answer all cannabis policy positions and priorities questions

^b^Participants were asked to list their top 3 priorities

^c^Participants were asked to list their top 3 priorities, except for “not allowing packaging attractive to youth,” which was answered separately

^d^Participants were asked to list their top 3 priorities

Elected officials were generally interested in being involved in cannabis industry regulation, regardless of their political party affiliation. Of the 185 who indicated their interest level, 75% reported being somewhat or very interested, and 59% believed cannabis policy is a priority.

### I. Positions on cannabis legalization in local jurisdictions

One hundred ninety-one elected officials responded to questions about their support for local retail legalization. Overall, the majority supported legalization in their local jurisdictions, including licensing medical retailers (56%), adult-use retailers (53%), and other business types (e.g., cultivation, manufacturing) (51%); less than half supported allowing delivery by retailers based outside their jurisdiction (41%). However, differences arose by political party (Table 3). Support for licensing medical retailers was significantly higher among Democrats (75%) than Republicans (42%; OR = 0.24 [0.11, 0.55]) and Independents (35%; OR = 0.18 [0.08, 0.42]). Similarly, support for adult-use retailers was significantly higher among Democrats (72%) than Republicans (39%; OR = 0.25 [0.11, 0.56]) and Independents (38%; OR = 0.23 [0.10, 0.53]). These differences were consistent among respondents in jurisdictions that banned retail (Table 4**)**; however, in jurisdictions that allowed cannabis retail, Democrats and Republicans did not significantly differ in their support of licensing medical retailers (OR = 0.86 [0.16, 4.73]), or adult-use retailers (D: 78%, R:70%; OR = 0.64 [0.14, 2.90]).

**Table 3:** Associations between cannabis policy positions and priorities and political affiliation (*n* = 155)

	Republicans vs. Democrats	Independents vs. Democrats	Independents vs. Republicans	*p*-value
	OR (95% CI)	OR (95% CI)	OR (95% CI)	
Experience in cannabis policy				
Ever had direct involvement in cannabis policy in California	0.88 (0.39, 1.94)	1.14 (0.52, 2.52)	1.31 (0.52, 3.30)	0.852
Policy issue experience				
Taxation	0.39 (0.14, 1.05)	0.40 (0.15, 1.08)	1.03 (0.30, 3.55)	0.055
Implementing rules for business operations	0.60 (0.23, 1.55)	0.85 (0.35, 2.08)	1.42 (0.47, 4.33)	0.552
Establishment of legal commerce	0.68 (0.26, 1.78)	1.27 (0.53, 3.02)	1.87 (0.64, 5.53)	0.511
Use of tax revenue	0.78 (0.26, 2.38)	1.66 (0.64, 4.32)	2.12 (0.64, 7.07)	0.425
Eliminating the illicit market	1.06 (0.30, 3.76)	1.41 (0.43, 4.63)	1.33 (0.33, 5.39)	0.853
Public education	0.74 (0.22, 2.49)	1.46 (0.52, 4.14)	1.98 (0.53, 7.45)	0.579
Equity in licensing	0.09 (0.00, 1.53)	1.14 (0.37, 3.46)	13.03 (0.69, 244.64)	0.224^c^
Opposition to legal commerce	5.91 (1.09, 32.01)	1.08 (0.10, 12.34)	0.18 (0.02, 1.65)	0.064
Cannabis use prevention or treatment	0.13 (0.01, 2.29)	1.32 (0.38, 4.54)	10.34 (0.54, 199.25)	0.302^c^
Marketing	0.29 (0.01, 5.71)	0.30 (0.01, 5.86)	1.03 (0.02, 53.09)	0.560^c^
Interest in cannabis industry regulation				
Somewhat or very interested in being involved	0.94 (0.38, 2.33)	0.60 (0.25, 1.44)	0.65 (0.23, 1.79)	0.513
Cannabis policy is priority	0.63 (0.29, 1.39)	0.48 (0.22, 1.07)	0.77 (0.31, 1.90)	0.166
Support for cannabis legalization in local jurisdictions				
Licensing of medical retailers	**0.24 (0.11**,** 0.55)**	**0.18 (0.08**,** 0.42)**	0.74 (0.29, 1.89)	< 0.001
Licensing of adult-use retailers	**0.25 (0.11**,** 0.56)**	**0.23 (0.10**,** 0.53)**	0.93 (0.37, 2.37)	< 0.001
Licensing other non-retailer business types	0.46 (0.21, 1.01)	0.54 (0.24, 1.18)	1.17 (0.47, 2.91)	0.093
Allowing outside jurisdiction delivery	**0.29 (0.13**,** 0.68)**	0.44 (0.20, 0.99)	1.52 (0.56, 4.08)	0.006
Approached by stakeholders (% yes)				
Private citizens	1.06 (0.49, 2.31)	1.01 (0.46, 2.21)	0.95 (0.38, 2.36)	0.988
Cannabis industry representatives	1.01 (0.46, 2.20)	0.96 (0.44, 2.10)	0.95 (0.38, 2.36)	0.994
Most important cannabis policy issues among elected officials^a^				
Tax revenue	0.73 (0.33, 1.58)	0.61 (0.27, 1.35)	0.84 (0.33, 2.11)	0.426
Economic development	0.60 (0.27, 1.35)	0.63 (0.28, 1.41)	1.04 (0.40, 2.70)	0.338
Youth cannabis use	1.51 (0.68, 3.35)	1.97 (0.89, 4.36)	1.30 (0.52, 3.24)	0.221
Eliminating the illicit market	1.52 (0.68, 3.41)	1.12 (0.48, 2.59)	0.74 (0.29, 1.90)	0.596
Adverse health or social effects of cannabis use	**3.10 (1.36**,** 7.07)**	1.28 (0.52, 3.12)	0.41 (0.16, 1.08)	0.024
Environmental issues	1.20 (0.37, 3.85)	0.96 (0.27, 3.33)	0.80 (0.20, 3.25)	0.942
Social equity	**0.11 (0.01**,** 0.85)**	**0.23 (0.05**,** 1.05)**	2.11 (0.18, 24.37)	0.005
Criminal justice	0.29 (0.06, 1.34)	0.62 (0.19, 2.07)	2.18 (0.37, 12.71)	0.192
Most important cannabis policy issues among constituents^a^				
Negative health impacts of youth use	1.05 (0.48, 2.27)	0.49 (0.21, 1.13)	0.47 (0.18, 1.22)	0.184
Eliminating the illicit market	0.63 (0.27, 1.48)	0.95 (0.42, 2.15)	1.52 (0.56, 4.08)	0.541
Negative community impacts of cannabis business	2.23 (1.00, 4.96)	1.51 (0.66, 3.43)	0.68 (0.27, 1.70)	0.137
Negative health impacts of cannabis consumption	**4.10 (1.56**,** 10.76)**	0.70 (0.18, 2.74)	**0.17 (0.04**,** 0.66)**	0.004
Negative social impacts of cannabis use, such as on work or education	3.24 (1.10, 9.51)	1.26 (0.35, 4.62)	0.39 (0.11, 1.40)	0.092
Most effective regulatory actions for youth prevention^b^				
Not allowing packaging attractive to youth	0.73 (0.22, 2.41)	0.30 (0.11, 0.84)	0.41 (0.12, 1.34)	0.068
Limiting products mimicking candies or foods attractive to youth	0.78 (0.36, 1.71)	0.60 (0.27, 1.32)	0.77 (0.31, 1.90)	0.432
Eliminating the illicit market	0.91 (0.42, 1.97)	0.96 (0.44, 2.10)	1.06 (0.43, 2.62)	0.971
Limiting/prohibiting flavored inhaled products	0.51 (0.23, 1.15)	0.60 (0.27, 1.34)	1.17 (0.45, 3.07)	0.188
Using tax revenue for youth programs	**0.23 (0.06**,** 0.81)**	0.62 (0.24, 1.60)	2.72 (0.65, 11.46)	0.032
Education campaigns	0.61 (0.18, 2.00)	1.20 (0.44, 3.32)	1.98 (0.53, 7.45)	0.566
Establishing larger buffer zones from retailers	**0.22 (0.05**,** 1.02)**	0.62 (0.21, 1.86)	2.81 (0.51, 15.51)	0.077
Priority uses of cannabis tax revenue^a^				
General fund with spending flexibility	0.60 (0.27, 1.31)	0.57 (0.26, 1.25)	0.95 (0.38, 2.34)	0.253
Law enforcement	**6.47 (2.75**,** 15.23)**	2.01 (0.89, 4.54)	**0.31 (0.12**,** 0.81)**	< 0.001
Community reinvestment	0.64 (0.28, 1.48)	0.51 (0.21, 1.22)	0.79 (0.28, 2.20)	0.249
Public education on cannabis or substance use disorder	0.74 (0.31, 1.75)	1.26 (0.56, 2.85)	1.70 (0.64, 4.55)	0.562
Preventing substance use disorder	0.68 (0.26, 1.78)	1.11 (0.46, 2.69)	1.64 (0.55, 4.90)	0.634
Supporting vulnerable youth	**0.29 (0.09**,** 0.91)**	0.58 (0.22, 1.50)	1.98 (0.53, 7.45)	0.060

*p*-values < 0.05 after applying the Bonferroni correction indicated in bold. Among those who identified their political affiliation, represented one jurisdiction (i.e., city or county only) and answered all questions, there were 80 Democrats, 38 Republicans, and 37 Independents. P-values indicate statistical significance of overall association between response endorsement and political affiliation based on likelihood ratio tests derived from logistic regressions

^a^Participants selected their top 3 responses

^b^Participants selected their top 3 responses with the exception of “not allowing packaging attractive to youth,” which was answered separately

^c^Firth logistic regression and Wald tests were used

**Table 4:** Associations between cannabis policy positions and priorities and political affiliation, by cannabis retail policy (*n* = 155)

	Among retail-banning jurisdictions (*n* = 73)				Among retail-allowing jurisdictions (*n* = 82)			
	Republicans vs. Democrats	Independents vs. Democrats	Independents vs. Republicans	p-Value	Republicans vs. Democrats	Independents vs. Democrats	Independents vs. Republicans	p-Value
	OR (95% CI)	OR (95% CI)	OR (95% CI)		OR (95% CI)	OR (95% CI)	OR (95% CI)	
Experience in cannabis policy								
Ever had direct involvement in cannabis policy in California	1.24 (0.40, 3.88)	1.19 (0.31, 4.53)	0.96 (0.26, 3.60)	0.926	1.12 (0.29, 4.37)	1.24 (0.45, 3.42)	1.10 (0.24, 4.96)	0.916
Policy issue experience								
Taxation	0.24 (0.04, 1.60)	0.13 (0.01, 2.61)	0.56 (0.02, 14.45)	0.183^c^	1.43 (0.37, 5.56)	0.57 (0.19, 1.71)	0.40 (0.08, 1.90)	0.450
Implementing rules for business operations	0.48 (0.08, 2.86)	0.89 (0.14, 5.51)	1.86 (0.24, 14.64)	0.694	1.83 (0.47, 7.19)	0.92 (0.31, 2.68)	0.50 (0.11, 2.32)	0.644
Establishment of legal commerce	0.29 (0.05, 1.61)	0.88 (0.19, 4.14)	3.00 (0.44, 20.24)	0.291	2.64 (0.66, 10.54)	1.63 (0.56, 4.76)	0.62 (0.13, 2.82)	0.332
Use of tax revenue	0.13 (0.01, 2.69)	0.73 (0.10, 5.48)	5.52 (0.21, 143.67)	0.421^c^	4.10 (0.99, 16.95)	2.52 (0.82, 7.73)	0.62 (0.13, 2.82)	0.079
Eliminating the illicit market	0.67 (0.10, 4.33)	0.58 (0.06, 6.06)	0.87 (0.07, 10.38)	0.863	2.30 (0.38, 13.96)	2.16 (0.52, 9.02)	0.94 (0.14, 6.25)	0.479
Public education	0.48 (0.08, 2.86)	0.42 (0.04, 4.09)	0.87 (0.07, 10.38)	0.616	1.57 (0.28, 8.98)	2.51 (0.73, 8.67)	1.60 (0.26, 9.83)	0.350
Equity in licensing	0.13 (0.01, 2.69)	0.23 (0.01, 4.73)	1.73 (0.03, 91.20)	0.311^c^	0.28 (0.01, 5.35)	1.98 (0.57, 6.81)	7.00 (0.35, 140.13)	0.327^c^
Opposition to legal commerce	4.67 (0.49, 44.64)	1.87 (0.11, 32.01)	0.40 (0.04, 3.93)	0.312	5.32 (0.50, 56.69)	0.78 (0.03, 19.99)	0.15 (0.01, 3.96)	0.308^c^
Cannabis use prevention or treatment	0.19 (0.01, 4.20)	1.90 (0.29, 12.24)	9.83 (0.44, 218.48)	0.343^c^	0.40 (0.02, 7.86)	1.08 (0.22, 5.29)	2.69 (0.12, 61.44)	0.818^c^
Marketing	0.19 (0.01, 4.20)	0.33 (0.02, 7.38)	1.73 (0.03, 91.20)	0.505^c^	1.60 (0.06, 42.14)	0.78 (0.03, 19.99)	0.49 (0.01, 26.37)	0.937^c^
Interest in cannabis industry regulation								
Somewhat or very interested in being involved	0.95 (0.31, 2.90)	0.58 (0.16, 2.05)	0.61 (0.17, 2.16)	0.667	4.69 (0.25, 87.29)	0.67 (0.20, 2.21)	0.14 (0.01, 2.86)	0.420^c^
Cannabis policy is priority	0.70 (0.25, 2.00)	0.27 (0.07, 1.04)	0.38 (0.10, 1.49)	0.138	1.51 (0.29, 8.02)	0.76 (0.25, 2.26)	0.50 (0.08, 3.01)	0.729
Support for cannabis legalization in local jurisdictions								
Licensing of medical retailers	**0.24 (0.08**,** 0.74)**	**0.20 (0.05**,** 0.79)**	0.83 (0.21, 3.37)	0.012	0.86 (0.16, 4.73)	**0.16 (0.05**,** 0.49)**	0.19 (0.03, 1.11)	0.004
Licensing of adult-use retailers	**0.24 (0.08**,** 0.74)**	**0.20 (0.05**,** 0.79)**	0.83 (0.21, 3.37)	0.012	0.64 (0.14, 2.90)	**0.25 (0.08**,** 0.74)**	0.39 (0.08, 1.93)	0.041
Licensing other non-retailer business types	0.29 (0.09, 0.93)	0.49 (0.13, 1.76)	1.67 (0.42, 6.69)	0.096	9.76 (0.54, 176.77)	0.61 (0.22, 1.71)	0.06 (0.00, 1.21)	0.162^c^
Allowing outside jurisdiction delivery	**0.15 (0.05**,** 0.52)**	**0.16 (0.04**,** 0.70)**	1.06 (0.22, 5.18)	0.002	0.89 (0.23, 3.45)	0.81 (0.29, 2.24)	0.91 (0.20, 4.10)	0.916
Approached by stakeholders (% yes)								
Private citizens	1.42 (0.49, 4.08)	0.55 (0.14, 2.12)	0.38 (0.10, 1.49)	0.361	2.37 (0.46, 12.37)	1.90 (0.60, 6.02)	0.80 (0.13, 5.07)	0.362
Cannabis industry representatives	1.07 (0.38, 3.03)	0.74 (0.21, 2.57)	0.69 (0.20, 2.43)	0.839	2.58 (0.50, 13.41)	1.29 (0.44, 3.75)	0.50 (0.08, 3.01)	0.473
Most important cannabis policy issues among elected officials^a^								
Tax revenue	0.65 (0.22, 1.99)	1.27 (0.37, 4.40)	1.94 (0.54, 7.02)	0.565	3.03 (0.59, 15.73)	0.38 (0.13, 1.10)	**0.13 (0.02**,** 0.75)**	0.034
Economic development	0.47 (0.15, 1.46)	0.64 (0.18, 2.34)	1.36 (0.35, 5.31)	0.415	1.56 (0.39, 6.20)	0.64 (0.23, 1.81)	0.41 (0.09, 1.92)	0.489
Youth cannabis use	1.90 (0.65, 5.51)	1.90 (0.55, 6.59)	1.00 (0.29, 3.42)	0.422	0.55 (0.10, 2.87)	1.99 (0.70, 5.63)	3.64 (0.62, 21.36)	0.251
Eliminating the illicit market	1.49 (0.47, 4.76)	1.05 (0.25, 4.32)	0.70 (0.18, 2.80)	0.774	3.00 (0.75, 12.08)	1.23 (0.43, 3.54)	0.41 (0.09, 1.92)	0.293
Adverse health or social effects of cannabis use	3.43 (1.15, 10.24)	1.01 (0.27, 3.77)	0.29 (0.08, 1.08)	0.045	0.52 (0.06, 4.62)	1.46 (0.42, 5.02)	2.81 (0.28, 27.97)	0.623
Environmental issues	1.88 (0.40, 8.77)	2.00 (0.35, 11.32)	1.06 (0.22, 5.18)	0.644	0.33 (0.02, 6.39)	0.51 (0.08, 3.26)	1.54 (0.06, 41.08)	0.631^c^
Social equity	0.06 (0.00, 1.19)	0.11 (0.01, 2.08)	1.73 (0.03, 91.20)	0.077^c^	0.46 (0.05, 4.02)	0.43 (0.09, 2.16)	0.95 (0.08, 11.87)	0.469
Criminal justice	0.18 (0.02, 1.63)	0.32 (0.03, 3.01)	1.80 (0.10, 30.89)	0.187	0.60 (0.07, 5.39)	0.90 (0.21, 3.77)	1.50 (0.14, 16.54)	0.889
Most important cannabis policy issues among constituents^a^								
Negative health impacts of youth use	0.81 (0.29, 2.29)	0.42 (0.12, 1.53)	0.52 (0.14, 1.91)	0.404	1.32 (0.34, 5.12)	0.53 (0.18, 1.58)	0.40 (0.08, 1.90)	0.405
Eliminating the illicit market	1.04 (0.27, 4.09)	2.18 (0.52, 9.12)	2.09 (0.50, 8.76)	0.511	1.12 (0.29, 4.37)	0.69 (0.25, 1.96)	0.62 (0.13, 2.82)	0.742
Negative community impacts of cannabis business	3.50 (1.16, 10.58)	3.37 (0.94, 12.14)	0.96 (0.28, 3.33)	0.045	0.60 (0.11, 3.16)	0.75 (0.23, 2.42)	1.25 (0.20, 7.92)	0.770
Negative health impacts of cannabis consumption	3.37 (1.04, 11.00)	0.13 (0.01, 2.61)	0.04 (0.00, 0.73)	0.022^c^	1.31 (0.13, 13.08)	1.96 (0.40, 9.63)	1.50 (0.14, 16.54)	0.719
Negative social impacts of cannabis use, such as on work or education	4.50 (0.85, 23.95)	1.93 (0.24, 15.18)	0.43 (0.08, 2.37)	0.151	2.30 (0.38, 13.96)	0.97 (0.17, 5.43)	0.42 (0.05, 3.53)	0.667
Most effective regulatory actions for youth prevention^b^								
Not allowing packaging attractive to youth	0.74 (0.18, 3.08)	0.21 (0.05, 0.87)	0.28 (0.07, 1.11)	0.071	1.99 (0.10, 39.85)	0.50 (0.11, 2.24)	0.25 (0.01, 5.36)	0.536^c^
Limiting/prohibiting products mimicking candies or foods attractive to youth	0.45 (0.15, 1.33)	0.45 (0.13, 1.58)	1.00 (0.29, 3.42)	0.271	1.77 (0.41, 7.63)	0.69 (0.25, 1.91)	0.39 (0.08, 1.93)	0.488
Eliminating the illicit market	0.93 (0.33, 2.64)	0.93 (0.28, 3.16)	1.00 (0.29, 3.42)	0.990	1.14 (0.29, 4.53)	1.01 (0.36, 2.82)	0.89 (0.19, 4.11)	0.983
Limiting/prohibiting flavored inhaled products	0.29 (0.09, 0.93)	0.83 (0.24, 2.84)	2.85 (0.75, 10.87)	0.082	1.69 (0.42, 6.70)	0.45 (0.15, 1.34)	0.27 (0.05, 1.29)	0.188
Using tax revenue for youth programs	0.29 (0.05, 1.61)	0.26 (0.03, 2.34)	0.87 (0.07, 10.38)	0.217	0.24 (0.03, 2.08)	0.87 (0.29, 2.67)	3.60 (0.37, 34.94)	0.326
Education campaigns	0.58 (0.12, 2.68)	0.32 (0.03, 3.01)	0.56 (0.05, 5.84)	0.520	0.60 (0.07, 5.39)	2.15 (0.64, 7.22)	3.60 (0.37, 34.94)	0.352
Establishing larger buffer zones from retailers	**0.17 (0.03**,** 0.88)**	0.32 (0.06, 1.70)	1.86 (0.24, 14.64)	0.050	0.28 (0.01, 5.35)	1.12 (0.28, 4.46)	3.97 (0.19, 84.60)	0.672^c^
Priority uses of cannabis tax revenue^a^								
General fund with spending flexibility	0.46 (0.16, 1.33)	0.53 (0.15, 1.83)	1.15 (0.34, 3.95)	0.315	0.97 (0.24, 3.86)	0.59 (0.21, 1.63)	0.61 (0.13, 2.79)	0.583
Law enforcement	**4.06 (1.33**,** 12.34)**	3.37 (0.94, 12.14)	0.83 (0.24, 2.89)	0.027	**54.31 (2.99**,** 988.09)**	1.34 (0.46, 3.90)	**0.02 (0.00**,** 0.48)**	0.026^c^
Community reinvestment	0.57 (0.16, 2.02)	0.38 (0.07, 2.03)	0.66 (0.11, 3.86)	0.436	1.83 (0.46, 7.26)	0.61 (0.21, 1.76)	0.33 (0.07, 1.58)	0.359
Public education on cannabis or substance use disorder	0.52 (0.16, 1.69)	0.63 (0.16, 2.48)	1.22 (0.29, 5.20)	0.529	1.46 (0.36, 5.89)	1.99 (0.70, 5.63)	1.36 (0.30, 6.28)	0.422
Preventing substance use disorder	0.57 (0.16, 2.02)	0.88 (0.22, 3.53)	1.53 (0.35, 6.79)	0.669	0.81 (0.15, 4.36)	1.30 (0.41, 4.09)	1.60 (0.26, 9.83)	0.851
Supporting vulnerable youth	0.64 (0.16, 2.56)	0.88 (0.19, 4.14)	1.38 (0.27, 7.15)	0.811	0.09 (0.01, 1.70)	0.51 (0.16, 1.66)	5.40 (0.26, 110.68)	0.173^c^

*p*-values < 0.05 after applying the Bonferroni correction indicated in bold. Among participants in retail-banning jurisdictions, there were 29 Democrats, 28 Republicans, and 16 Independents. Among participants in retail-allowing jurisdictions, there were 51 Democrats, 10 Republicans, and 21 Independents. *P*-values indicate statistical significance of overall association between response endorsement and political affiliation based on likelihood ratio tests derived from logistic regressions, stratified by whether all forms of cannabis retail were banned vs. any were allowed as of January 1, 2024

^a^Participants selected their top 3 responses

^b^Participants selected their top 3 responses with the exception of “not allowing packaging attractive to youth,” which was answered separately

^c^Firth logistic regression and Wald tests were used

Republicans were less likely to support allowing businesses from outside their jurisdiction to deliver within their jurisdiction (26%) compared to Democrats (55%; OR = 0.29 [0.13, 0.68]), but Independents did not significantly differ in their support of outside delivery businesses (35%) from the other two political affiliations (Table 3). When stratifying by their local cannabis retail policy, a different pattern emerged: among jurisdictions that banned cannabis retail, the difference between Democrats (59%) and Republicans (18%; OR = 0.15 [0.05, 0.52]), as well as between Democrats and Independents (19%; OR = 0.16 [0.04, 0.70]), widened and was significantly different in each comparison (Table 4). In contrast, about half of respondents in jurisdictions that allowed cannabis retail supported outside delivery, with no significant variation across political affiliations (D: 53%, R: 50%, I: 48%; χ^2^(2) = 10.15, *p* = 0.916). Support for licensing other non-retailer business types was not significantly different by party overall or stratified by local cannabis retail policy.

#### A. Support and opposition for legal cannabis businesses

Local elected officials in the Superior California, San Francisco Bay Area, and North Coast regions described their support for cannabis legalization – for some, their support was related to their professional experiences working in the cannabis industry, illustrating both personal knowledge and potential conflicts of interest.

- “I was on the Board of Supervisors and helped write the ordinance that made it legal to grow, manufacture, and dispense cannabis [in this county]. It frustrates me that elected officials fail to realize that the industry is in its infancy and needs help working through a changing marketplace!” County Supervisor, San Francisco Bay Area.
- “I know cannabis policy very well and would be delighted to help in any way I can to improve policy moving forward.” City Council Member, Superior California.
- “I was the first City Council Member to push for our city to allow commercial cannabis sales.” City Council Member, San Francisco Bay Area.

City Council Members in the San Francisco Bay Area and Superior California communicated their reasons for supporting medical licenses but also highlighted concerns reflecting the differing realities across the state. They identified challenges associated with operating under a medical license within the broader cannabis market, ranging from insufficient local access to medical cannabis to market saturation.

- “It is not available in my town. I believe that my town would benefit from having a local dispensary. The current policy regarding cannabis in my area is much too strict and severely limits an individual’s ability to procure cannabis for medicinal purposes if they are unable to drive 25–30 minutes to purchase it.” City Council Member, Superior California.
- “I do agree there should be limited retail businesses for medical purposes, but the market is becoming saturated.” City Council Member, San Francisco Bay Area.

City Council Members also explained how their support for cannabis businesses had been complicated by litigation and over-regulation. One City Council Member in the Inland Empire proclaimed it is “time to stop regulating anything but storefronts.” Two elected officials in the North Coast Region described current activities related to improving local ordinances to help cannabis businesses:

- “The City Council has set cannabis ordinances as a top 10 priority with the expectation to develop about three retail stores in city limits. The legal department is working on this issue now.” City Council Member, North Coast.
- “We are currently working on combining and streamlining 10 separate cannabis-related ordinances.” County Supervisor, North Coast.

However, other elected officials in the North Coast and Central Coast critiqued cannabis legalization and argued for cannabis retail bans in their jurisdictions. In their words:

- “It should never have been legalized. Colorado has had nothing but problems since legalization, and now California has the same problems.” City Council Member, Central Coast.
- “No more retailers in our community. Sick of the drug area of cannabis.” City Council Member, North Coast.

Meanwhile, others emphasized that their communities face more urgent policy issues, neither communicating their support nor opposition for cannabis businesses; simply stating that cannabis policy does not rank as a top policy in their jurisdictions:

- “I am an elected official in a small city where housing issues are 100 times more important to my constituents relative to cannabis.” City Council Member, San Francisco Bay Area.
- “We have bigger issues in my county than cannabis: fentanyl use & sales, the homeless crisis, which is overwhelming ALL of my Departments, and zero future revenue ideas: just grants and other handouts.” County Supervisor, Superior California.

#### B. Stakeholder engagement

Elected officials across political parties reported being approached by stakeholders about cannabis policy issues. Of the 185 respondents who answered, over half had been approached by private citizens (56%) and representatives of the cannabis industry (54%). Concerns heard ranged from political pressure from those who stand to profit to preventing youth use and public safety. A City Council Member in San Diego-Imperial explained they had been approached by “parents asking for preventive measures.” A City Council Member in the San Francisco Bay Area encountered significant community resistance and concerns from local law enforcement, which influenced their decision to prohibit retail cannabis sales in their jurisdiction. “Given a lack of a County Cannabis Compliance force, lack of large support by our community (from a survey by the town), and opposition by our school districts, I realized this was not a fit for our town.”

In contrast, other City Council Members in the San Francisco Bay Area offered critical perspectives about stakeholder engagement in cannabis policy. They suggested that the stakeholders who had approached them about cannabis policy issues were driven solely by profit motives or had been influenced by others to support legal storefronts. In their words, they had been approached by “Only those trying to profit!” A second reported, “Senior citizens have been influenced to require local stores.”

#### C. Most effective regulatory actions for youth prevention

Banning packaging that is attractive to youth garnered cross-party support, with 84% (of the 191 elected officials who responded to the question) being supportive, regardless of political party affiliation. When asked to provide their top three most effective strategies for preventing youth use, over half reported limiting or prohibiting cannabis products mimicking candies or foods attractive to children and youth (e.g., Cocoa Pebbles, Skittles, Runtz) (55%) and eliminating the illicit cannabis market (53%). 40% agreed that limiting or prohibiting sales of flavored cannabis products for inhalation, like vapes (e.g., chocolate, mango, strawberry), was effective. In contrast, relatively few indicated that using cannabis tax revenue for youth programs (19%), establishing larger buffer zones between retailers and schools (16%), or education campaigns (16%) were among the most effective regulatory actions to reduce youth cannabis use. These findings did not differ by party affiliation, with two exceptions. A higher percentage of Democrats (28%), compared to Republicans (8%), positively viewed dedicating tax revenue for youth programs (OR = 0.23 [0.06, 0.81]) and establishing larger buffer zones for retailers (D: 20% vs. R: 5%; OR = 0.22 [0.05, 1.02]) as effective for youth prevention.

One City Council Member in the Inland Empire region emphasized the need to stop billboard advertising near schools: “Billboard advertising needs to discontinue. Just like cigarette advertising. Especially near schools.”

### II. Priority cannabis policy issues

In the overall sample, the top three cannabis policy priorities were tax revenue (41%), economic development (41%), and youth cannabis use (38%). Slightly fewer reported that eliminating the illicit market (34%) and the adverse health or social effects of cannabis use (30%) were among their top three most important cannabis policy issues. Least cited priorities included criminal justice (12%) and social equity (13%). However, the ranking of importance varied by political affiliation. Among Democrats, tax revenue (50%) and economic development (46%) were the most important, and youth cannabis use led among Independents (49%), though these differences were not significant. Whereas among Republicans, adverse health or social effects of cannabis use were significantly and three times more likely to be a top priority (47%) than among Democrats (22%; OR = 3.10 [1.36, 7.07]). Significant differences were also apparent in the lower-ranked issues. Social equity was prioritized by 20% of Democrats, but only 3% of Republicans (OR = 0.11 [0.01, 0.85]), and 5% of Independents (OR = 0.23 [0.05, 1.05]). These differences became non-significant when respondents were stratified by local cannabis retail policy.

Among the 191 elected officials who ranked the importance of cannabis policy issues for their constituents, the top three priorities were most often the negative health impacts of youth use (42%), eliminating the illicit market (36%), and negative community impacts of cannabis businesses (35%). However, Republicans were far more likely to report that the negative health impacts of cannabis consumption were a priority issue for their constituents (34%) than Democrats (11%; OR = 4.10 [1.56, 10.76]), and Independents (8%; OR = 0.17 [0.04, 0.66]) (χ^2^[2] = 7.59, *p* = 0.023).

#### Priority issues #1 and #2: tax revenue and economic development

Only one elected official provided a written response explaining why economic development and tax revenue were the top priority issues. Specifically, a City Council Member in the San Diego-Imperial region suggested cannabis tax revenue could be better utilized and offered a plan to speed up legalization across the State: “Cannabis is an underutilized tax resource. There should be a model for cities that may be more skeptical to adopt and implement easily. There should also be a shared space for elected leaders to share and learn online.”

#### Priority issue #3: youth use

Elected officials in the San Francisco Bay Area and San Diego-Imperial regions expressed their concerns about the negative health impacts of youth cannabis use, including reporting a rising incidence of associated serious adverse mental health issues. They highlighted how the medical cannabis market was facilitating greater youth access and articulated a series of specific health risks associated with youth use. They focused on the potency of cannabis and underscored how interests in profit potential and local tax revenue were facilitating a troubling situation for youth. Some also noted their concern that cannabis is a ‘gateway’ to other addictive drugs, including tobacco.

- “I have seen too many promising youth blow up their lives ever since.” City Council Member, Southern San Joaquin Valley.
- “I understand that teen use of marijuana is unavoidable, but the ease of access and potency are insane. Dependencies appear to develop quickly. I realize that something else would probably have filled that void, and I don’t know what it was like pre-legalization. Still, if there is a profit (tax collection) driven motivation to legalization, it is pretty f’d up.” City Council Member, San Francisco Bay Area.
- “With the open access to cannabis through medical use for several years now, we are seeing an increased incidence of mental health disease among 20–30-year-olds. It was known before legalization that youth with a predisposition to schizophrenia, depression, or bipolar disorder have a stronger incidence of converting to disease if they use cannabis to self-medicate. This has been a problem and is now emerging as an issue.” City Council Member, San Diego-Imperial.
- “I believe that cannabis is a gateway to other addictive drugs and leads our youth to smoking also.” City Council Member, San Francisco Bay Area.

#### Priority issue #4: eliminating the illicit market

Elected officials in the San Francisco Bay Area and Superior California regions reported their concerns about the illicit market, given its association with crime and drug trafficking, which have impacts on local communities. Multiple respondents reiterated that the illicit market was dangerous because illegal activities could pollute the cannabis supply. They emphasized the risks of ‘Black Market’ cannabis, which they believed could contain fentanyl, thus introducing lethal consequences for cannabis users, including for youth, who may be encountering cannabis containing fentanyl at schools. They also contended that the competitive presence of the illegal market was threatening the success of the legal market.

- “Until the illicit cannabis market can be controlled, the legal market will not be profitable and functional. Much of our serious crime is related to illegal cultivation and the black market.” County Supervisor, Superior California.
- “Black market cannabis is terrifying in today’s world. Legal cannabis businesses fail because of the competitive advantage given to black market businesses due to high taxation and over-regulation. Black market cannabis, legal cannabis that fails regulatory testing, and unregulated Delta-8 cannabis ends up being sold in high school bathrooms. This is the product that will contain fentanyl and will literally kill our youth. It is mind-boggling that preventing this is not everyone’s priority.” City Council Member, San Francisco Bay Area.
- “One of the emerging concerns I see is the prevalence of illegal cannabis being delivered to licensed facilities that contain pesticides that are either banned in California and/or fail the pesticide standards. Many people are consuming cannabis that does not meet the pesticide standards of the Department of Cannabis Control. Our agency has been testing illegal cannabis being exported to other areas, and it is failing the testing standards. This is concerning to me.” Sherriff, Superior California.

#### Priority issue #5: adverse health or social effects of cannabis use

Elected officials expressed concerns about both the adverse health and social effects of cannabis use. Most of their concerns lacked specific details about health or social impacts. However, some exhibited specific technical knowledge about health impacts and potential regulatory actions to help prevent problem use. Four City Council Members throughout the State expressed general concerns about the adverse health and social effects of legalization and of cannabis use, asserting legal cannabis was ‘dumbing down’ the nation, had a deleterious impact on the fabric of the community, negatively affected individual character and personality, and thus communicated the imperative to prevent the normalization of cannabis. These City Council Members framed the topic using political discourse reminiscent of the War on Drugs messaging:

- “The legalization of marijuana is the dumbing down of America.” City Council Member, Superior California.
- “My experience in South Vietnam convinced me that prolonged use of cannabis has a negative impact on one’s character and personality.” City Council Member, Los Angeles County.
- “We need to stop these types of drugs from being commonplace.” City Council Member, Los Angeles County.
- “Cannabis is only one government-sponsored cancer eating away at the fabric of our community.” City Council Member, Orange County.

Reflecting the experience of the views of the elected officials described above, a City Council Member in Orange County suggested, in contrast, that the fear of cannabis exhibited among other elected officials and members of their community is deeply seated in a past cultural moment – reminiscent of alcohol Prohibition (1920–1933) and the Reefer Madness era – and could be addressed through public education about the risks and benefits of cannabis:

- “Many residents and elected officials still harbor a ‘Reefer Madness’ type fear of cannabis. I believe that a State-level education program outlining accurate risks and benefits of cannabis would help alleviate some of the fear surrounding cannabis use.” City Council Member, Orange County.

A second City Council Member in Orange County exhibited specific knowledge about cannabis use-associated problems and suggested potential solutions to protect public health.

- “The increased use of edibles by new users is a leading cause of many negative experiences… Sales of edibles should include verbal and written guidance on appropriate dosage and timing.” City Council Member, Orange County.

However, other City Council Members were more supportive of legal cannabis, highlighting the positive health effects of cannabis and drawing a sharp contrast between the illegal and legal products:

- “I believe that cannabis has positive health effects.” City Council Member, Superior California.
- “Legal, regulated cannabis is not a dangerous drug.” City Council Member, San Francisco Bay Area.

#### Priority uses of cannabis tax revenue

When asked to rank their top three priorities for uses of cannabis tax revenue, the most common uses included: contributing to the general fund (with spending flexibility) (55%), law enforcement (40%), and community reinvestment (defined as small business development in vulnerable communities) (36%). Slightly fewer prioritized using cannabis tax revenue to provide public education on cannabis or substance use disorders (31%); and relatively few prioritized using cannabis tax revenue to prevent substance use disorders (23%), or to support vulnerable youth (22%), though Democrats were more likely to prioritize supporting vulnerable youth (29%) than Republicans (11%; OR = 0.29 [0.09, 0.91]).

Among Republicans, law enforcement was the most commonly top-ranked choice for uses of cannabis tax revenue (71%), whereas significantly fewer Democrats (28%; OR = 6.47 [2.75, 15.23]) and Independents (43%; OR = 0.31 [0.12, 0.81]) ranked it highly. Within jurisdictions that allowed retail, partisan divides widened, with an estimated 95% of Republicans ranking law enforcement as the best use for tax revenue compared to 28% of Democrats (OR = 54.31 [2.99, 988.09]) and 34% of Independents (OR = 0.02 [0.00, 0.48]). Within jurisdictions that banned cannabis retail, political differences decreased but were still significant between Democrats and Republicans (OR = 4.06 [1.33, 12.34]).

## Discussion

This study provides the first systematic evidence on how U.S. elected officials prioritize cannabis policy issues, with significant implications for understanding how local political dynamics may influence public health outcomes. Our findings illuminate the political context in which local regulatory decisions are made. These decisions directly influence cannabis availability, marketing environments, and ultimately public-level health outcomes [8, 12].

The prioritization of economic considerations, including tax revenue (41%) and economic development (41%), over explicit health concerns among California’s elected officials helps explain why, despite mounting evidence of cannabis-related health harms, few local jurisdictions have implemented robust public health protections. This pattern reflects broader dynamics in the political economy of public health, wherein commercial interests often outweigh health considerations in policy formation [49]. The cannabis industry, like tobacco and alcohol before it, has developed significant political influence over regulatory outcomes [50], and our finding that over half of elected officials had been approached by cannabis industry representatives suggests normalization of industry engagement in local policy processes.

### Cross-party agreement and public health opportunities

Despite deep partisan divides on foundational questions of legalization, we found notable cross-party agreement on several cannabis policy issues with direct implications for public health. The strongest cross-party support – among 84% of all elected officials in this sample – emerged for regulating commercial cannabis by prohibiting packaging attractive to youth. This consensus represents a significant opportunity for public health advocacy, as packaging regulations have proven effective in other contexts for reducing the youth appeal of harmful products [51]. Similarly, officials across party lines agreed on the importance of eliminating the illicit market (53%) and limiting products that mimic candies or foods attractive to children (55%), suggesting a shared concern for youth protection that transcends partisan divides.

Cross-party support signals opportunities for evidence-based policy change. However, our findings also reveal a troubling policy-practice gap: while 84% of officials supported youth-attractive packaging restrictions, only 12 of 320 California jurisdictions that allow retail sales have adopted regulations that improve upon the state’s vague limitation on content “attractive to individuals under the age of 21.” The disconnects between policymaker attitudes and regulatory action suggest that structural barriers, including industry lobbying, competing priorities, and limited technical capacity, may impede translation of health-protective preferences into policy. Understanding and addressing these barriers represents an essential agenda for public health research and practice.

### Health equity implications

The health equity implications of these findings are substantial. Social equity was prioritized by only 13% of elected officials overall, with significant partisan variation: 20% of Democrats and only 3% of Republicans ranked it among their top priorities. This low prioritization is concerning given evidence that cannabis retailers disproportionately concentrate in lower-income and minority communities [9], potentially exacerbating existing health disparities. Without explicit policy attention to equity, cannabis legalization may reproduce or amplify the unequal distribution of health harms that characterized prohibition-era enforcement [13].

The partisan differences in priority uses of cannabis tax revenue further illuminate potential equity implications. Republicans were significantly more likely to prioritize law enforcement (71%) compared to Democrats (28%), while Democrats were more likely to prioritize supporting vulnerable youth (29% vs. 11%). These divergent priorities may translate into different patterns of resource allocation across jurisdictions, with potential consequences for community health. In jurisdictions prioritizing enforcement, communities historically targeted by drug enforcement may continue to experience disproportionate criminal justice contact, with well-documented negative health consequences. Conversely, jurisdictions directing resources toward prevention and youth support may see different public health trajectories.

Our finding that partisan differences in support for licensing narrowed in jurisdictions that already allowed retail cannabis suggests a potential pathway for policy evolution. In these jurisdictions, Democrats and Republicans did not significantly differ in their support for medical or adult-use retail licensing, indicating that exposure to legal cannabis markets may reduce partisan polarization. However, this convergence may also reflect selection effects: jurisdictions that allow retail may have had less partisan division over cannabis policy from the outset. Longitudinal research is needed to understand how local cannabis policy environments evolve and their implications for health equity.

### Framing, evidence, and public health

The qualitative findings reveal diverse and sometimes conflicting framings of cannabis among elected officials, with implications for evidence-based policy. Some officials employed prohibition-era language portraying cannabis as the “dumbing down America” or “eating away at the fabric of our society.” In contrast, others described legal cannabis as “not a dangerous drug” with “positive health effects.” This polarization in framing reflects broader patterns documented by critical drug policy scholars, wherein drug policy is shaped as much by moral claims, political ideologies, and symbolic representations as by scientific evidence [30, 31, 35].

From a public health perspective, neither extreme framing aligns well with current scientific evidence. Cannabis use is associated with real health risks, particularly for youth, pregnant individuals, and those with predisposition to certain mental health conditions [1] but these risks are neither as catastrophic as prohibition-era narratives suggest nor as negligible as wellness-oriented framings imply. The persistence of these polarized frames among policymakers may impede the development of nuanced, evidence-based regulatory approaches that optimize public health outcomes. As one elected official noted, “many residents and elected officials still harbor a ‘Reefer Madness’ type fear of cannabis,” suggesting that educational interventions targeting policymakers may be warranted.

Notably, some officials demonstrated a sophisticated understanding of specific health risks and potential regulatory responses. One official highlighted concern about confusion over edible dosing, which has led to adverse experiences, and suggested that “sales of edibles should include verbal and written guidance on appropriate dosage and timing.” Another noted rising incidence of mental health conditions among young adults who used cannabis to self-medicate. These instances of health-informed policy thinking, while not dominant in our sample, suggest receptivity to evidence-based approaches among at least some policymakers.

### Local variation

California’s robust local control model creates conditions for substantial geographic variation in cannabis regulatory environments, with potential implications for public health inequities. Our findings demonstrate that policymaker priorities and positions vary significantly not only by political party but also by whether their jurisdictions already allow cannabis retail. This variation may produce a patchwork of regulatory approaches across the state, with different communities experiencing different levels of cannabis availability, marketing exposure, and health-protective regulation.

Indeed, research in California and elsewhere suggests that variations in local cannabis policies do affect public health outcomes. Local cannabis retail policies have been associated with cannabis use during pregnancy [11, 25], adolescent cannabis use patterns [11, 24], adverse adolescent mental health outcomes,[12] and poison control center contacts [8]. If policymaker priorities shape the regulatory environment, and the regulatory environment is strongly associated with health outcomes, then understanding local political dynamics is essential for predicting and potentially influencing public health trajectories. Our findings from California provide an initial foundation for this work by documenting the policy priorities and positions of elected officials in one large, locally controlled cannabis market. Replication in states with different regulatory structures, political climates, and market characteristics will be essential.

### Inconsistencies between stated priorities and policy action

Assessing elected officials’ priority uses of tax revenue provides insight into their true policy priorities beyond stated concerns. Our findings reveal notable inconsistencies: 38% of officials reported that youth cannabis use was among their most important policy issues, yet only 22% prioritized using cannabis tax revenue to support vulnerable youth, and only 23% prioritized prevention of substance use disorders. This gap between expressed concern and resource allocation preferences may reflect competing priorities, skepticism about intervention effectiveness, or the dominance of other considerations in budgetary decisions.

These inconsistencies mirror patterns observed in actual policy implementation across California. Nearly half of jurisdictions allowing cannabis activity do not levy local taxes, and fewer than one in ten dedicate revenue specifically to youth services or prevention [43]. The alignment between our survey findings and observed policy patterns suggests that elected officials’ stated priorities do not translate into policy action. For public health advocates, this suggests that simply raising awareness of youth cannabis risks may be insufficient; advocacy may need to address the competing priorities and structural factors that prevent concern from translating into action.

### Limitations

Several limitations should be considered when interpreting these findings. First, although our 9.3% response rate is consistent with challenges documented in policymaker survey research [44], selection bias may affect generalizability. Officials who responded may differ systematically from non-respondents in their engagement with or attitudes toward cannabis policy. Second, 36% of respondents did not report political party affiliation, potentially affecting the representativeness of our partisan analyses. Third, our survey focused on cannabis legalization and closely adjacent issues; we did not assess officials’ knowledge of recent health evidence or their broader policy priorities, which could provide additional context for understanding their cannabis-specific positions.

Fourth, our data reflect elected officials’ stated positions, which may or may not align with their actual voting behavior or policy decisions. Social desirability bias may influence responses, although the anonymous nature of the survey was designed to minimize this concern. Indeed, our finding that officials were willing to express positions contrasting with their jurisdictions’ official policies suggests some success in eliciting candid responses. Fifth, this study was conducted in California, the world’s largest legal cannabis market with robust local control. While this context provides a valuable case study, findings may not generalize to states with different regulatory structures, political climates, or cannabis market characteristics. Finally, the cross-sectional design limits our ability to understand how policymaker positions evolve over time or how they translate into regulatory outcomes.

## Conclusion

This study provides the first systematic examination of cannabis policy priorities and positions among elected officials in the United States, with significant implications for public health. Our findings document a political landscape in which economic considerations predominate over health concerns, partisan divides are evident in positions on foundational questions of legalization and resource allocation, and substantial gaps exist between policymaker attitudes toward health-protective measures and actual policy implementation. These dynamics may help explain why, despite growing evidence of cannabis-related health harms, robust public health protections remain rare in local cannabis regulatory environments.

However, our findings also identify opportunities for public health engagement. The strong cross-party agreement (84%) on restricting youth-attractive packaging represents an actionable consensus that could be leveraged for policy change. Similarly, widespread concern about youth cannabis use, even if inconsistently translated into resource allocation, suggests receptivity to evidence-based youth protection strategies. Public health practitioners and researchers should capitalize on these openings while working to elevate health equity considerations that currently receive limited attention in local cannabis policy discourse.

Looking forward, a robust public health approach to cannabis policy requires attention to the political dynamics documented here in these California policymakers. Research should examine whether and how policymakers’ priorities translate into regulatory outcomes, how those outcomes affect public health at the local level, and what interventions can effectively shift political priorities toward health protection and equity. Equally important is engagement with the diverse framings of cannabis held by elected officials that influence which policy options are considered legitimate and feasible. Strategic science – research deliberately designed to produce evidence that directly addresses the questions policymakers must answer, in formats and timeframes useful to policy decision-making [29] – offers a framework for bridging this gap. Strategic science can examine how local cannabis policy decisions are influenced by moral claims, economic interests, legacies of criminalization, and shifting cultural narratives, not only by evidence. Effective translation of evidence into policy will require understanding and engaging with these broader social and political forces.

In an era of rapid cannabis policy change across the United States, understanding policymakers’ perspectives is essential for protecting public health. The elected officials surveyed here are making decisions that will determine public health outcomes for years to come. Their priorities, positions, and framings matter. By documenting these perspectives and identifying opportunities for engagement, this study provides a starting foundation for evidence-informed advocacy and for building a more robust strategic science approach to cannabis policy that centers on public health and health equity.

## Supplementary Information

Supplementary Material 1

## Data Availability

The data that support the findings of this study are available from the corresponding author upon reasonable request.
