# Supplementary material for: Cannabis policy priorities and public health: a survey of elected officials in California
Source: BMC Public Health. 2026 May 29;26:2281. doi: 10.1186/s12889-026-27889-x (PMC13439760; doi:10.1186/s12889-026-27889-x)
Supplement: Supplementary file 1 [file 12889_2026_27889_MOESM1_ESM.docx]

**Supplementary File**

*This survey was developed specifically for this study. It has not been published elsewhere.*

**Assessing Engagement in Cannabis Policy: A Baseline Survey of Elected Officials in California**

**Introduction**

Thank you for agreeing to participate in this survey on cannabis policy in California. I appreciate your time and interest.

To begin, please verify some basic background information, and remember that this information will be held confidentially and used strictly for research purposes.

1. Are you an elected official?
   a. Yes, County District Attorney
   b. Yes, County Sheriff
   c. Yes, member of the Board of Supervisors
   d. Yes, member of the City Council
   e. Yes, member of the State Legislature
   f. No, but I am responding on behalf of an elected official
   g. No, I am not an elected official, nor am I responding on behalf of one

[If f, go to question 2; If g, end survey]

2. Who are you responding on behalf of?
   a. Member of the Board of Supervisors
   b. Member of the City Council
   c. Member of the State Legislature
   d. County District Attorney
   e. County Sheriff
   f. I am not representing an elected official.

[If f, end survey]

3. Are retail sales legal in your jurisdiction?
   a. Yes
   b. No
4. Have you ever been directly involved in work related to cannabis policy in California?
   a. Yes
   b. No
5. Which cannabis policy issue(s) have you (or your team) worked on? Please select all that apply.
   a. Cannabis use prevention or treatment
   b. Constraining the illicit market
   c. Equity in licensing
   d. Establishment of legal commerce
   e. Implementing rules for business operations
   f. Marketing
   g. Opposition to legal commerce
   h. Public education
   i. Taxation
   j. Use of tax revenue
   k. Other (please specify)
   l. None of the above
6. To what extent have the following factors posed barriers to your involvement in working on cannabis policy? A lack of ...
   a. Knowledge of cannabis policy best practices
   b. Knowledge of cannabis, its uses and effects
   c. Knowledge of priorities for communities
   d. Legal restrictions on proposing policy
   e. Political organization leadership and support
   f. Staff time for work on relevant issues
   g. Stakeholder support
   h. Technical resources or support
7. Which cannabis-related issues are most important to you? Please select up to three (3) issues from the list below.
   a. Adverse health or social effects of cannabis use
   b. Cannabis-associated crime or violence
   c. Criminal justice reform
   d. Economic development
   e. Environmental issues
   f. Health benefits of cannabis use
   g. Illicit market
   h. Social equity
   i. Tax revenue
   j. Youth use of cannabis
   k. Other (please specify)
8. Which cannabis-related issues are most important to your constituents? Please select up to three (3) issues from the list below.
   a. Access to regulated cannabis
   b. Business opportunities
   c. Eliminating the illegal market
   d. Equity in business opportunities
   e. Increased access to cannabis for medical use
   f. Negative community impacts of cannabis business
   g. Negative health impacts of cannabis consumption -general/all/any consumption
   h. Negative health impacts of cannabis consumption – heavy use
   i. Negative health impacts of youth use
   j. Negative impacts of cannabis-related crime and violence
   k. Negative social impacts of cannabis use, such as on work or education
   l. Reducing effects of criminalization
   m. Other (please specify)
   n. Don't know
9. Do you support or oppose the following approaches to cannabis policy?
   a. Allowing delivery of cannabis to your constituents from retailers located outside of your jurisdiction.
   b. Licensing of adult-use retailers (storefronts or deliverers) in your jurisdiction
   c. Licensing of medical retailers (storefronts or deliverers) in your jurisdiction
   d. Licensing of other business types (e.g., cultivation, manufacturing, distribution or testing) in your jurisdiction
   e. Not allowing packaging attractive to youth
10. Tax revenue from cannabis sales can used for different purposes. Should tax revenue be used in these ways? Please select up to three (3) potential uses of tax revenue from the list below.
    a. Community reinvestment (e.g., small business development in vulnerable communities)
    b. Environmental justice
    c. General fund with spending flexibility
    d. Law enforcement
    e. Mitigating harms of criminalization
    f. Preventing substance use disorder
    g. Public education on cannabis or substance use disorder
    h. Supporting vulnerable youth
    i. Treating substance use disorder
    j. Other (please specify)
11. Please indicate your level of concern about the adverse health effects of cannabis consumption.
    a. Very concerned
    b. Somewhat concerned
    c. Neutral
    d. Somewhat unconcerned
    e. Very unconcerned
    f. Don’t know
12. Which of the following regulatory actions would most effectively reduce cannabis use by youth? Please select up to three (3) regulatory actions.
    a. Advertising limits
    b. Capping the number of retailers
    c. Education campaigns
    d. Eliminating the illicit cannabis market Limiting or prohibiting sales of flavored cannabis products for inhalation, like vapes (e.g., chocolate, mango, strawberry)
    e. Establishing larger buffer zones (e.g., 1000 ft) from retailers to schools and other locations serving children or youth
    f. Increasing fines or penalties for youth possession
    g. Limiting or prohibiting cannabis products mimicking candies or foods attractive to children and youth (e.g., CocoaPebbles, Zkittles, Runtz)
    h. Limiting or prohibiting sales of high-potency products
    i. Limiting or prohibiting sales of intoxicating hemp products sold outside the legal cannabis system (e.g., Delta-8 THC gummies in smoke shops)
    j. Plain packaging (no images or graphics allowed on the package)
    k. Prohibiting delivery of cannabis
    l. Prohibiting storefront cannabis retailers
    m. Taxing higher potency (THC) products more (because they may post higher health risks)
    n. Using tax revenue for law enforcement
    o. Using tax revenue for youth programs
13. Please indicate your level of interest in being involved in the regulation of the cannabis industry.
    a. Very interested
    b. Somewhat interested
    c. Not interested
14. Is cannabis policy a priority for you? Please select the perspective that best matches your own.
    a. Yes, I believe cannabis is an important policy issue.
    b. No, I am not convinced cannabis is an important policy issue.
    c. I do not know enough about cannabis to consider it a policy priority.
15. Are you interested in doing additional work on any of the following cannabis policy areas? Please select up to three (3) cannabis policy areas from the list below.
    a. Business (e.g., licensing systems and operations)
    b. Constraining the illicit market
    c. Equity in licensing
    d. Marketing restrictions
    e. Opposition to legal sales
    f. Requiring information on health and safer use for consumers
    g. Restricting intoxicating hemp compounds (e.g., Delta-8 THC gummies in smoke shops)
    h. Restricting product types that are attractive to kids
    i. Restricting product types that are particularly harmful to users
    j. Substance use prevention
    k. Support for legal sales
    l. Taxation
    m. Use of tax revenue
    n. Other (please specify)
    o. None of the above
16. Which stakeholders/entities have approached you about cannabis-related issues? Please select all that apply.
    a. Colleges and Universities
    b. Community leaders
    c. Faith-based organizations
    d. Healthcare providers
    e. K-12 educators
    f. Law enforcement
    g. Mental health care providers
    h. Other elected officials
    i. Private citizens
    j. Public health officials
    k. Representatives of the cannabis industry
    l. Other (please specify)
    m. None of the above
17. Which political party are you affiliated with?
    a. Democratic
    b. Republican
    c. Independent
    d. No affiliation
    e. Other (please specify)
    f. Prefer not to answer
18. Are you familiar with the following technical resources to support local government work on cannabis policy? Please select all that apply. For reference, a project at the Public Health Institute publishes these resources and makes them freely available. On the next page, you'll have an option to request these technical resources and/or technical assistance.
    a. Not familiar
    b. California Cannabis Policy Scorecards evaluating cannabis rules and regulations for each local jurisdiction
    c. Model Ordinances on Cannabis Retail and Marketing in California
    d. Model Ordinances on General Tax for cannabis in California
    e. Model Ordinances on Special Taxes for cannabis in California
19. Would you like to share more information on your priorities for cannabis policy? I welcome your thoughts in the box below.
20. Please indicate your interest in the following items by checking the boxes below:
    a. I want to join a Listserv where I can receive more information about cannabis policy and take part in conversations about relevant issues.
    b. I want free technical assistance and/or resources to support work on cannabis policy in our community.
    c. I would consider participating in a follow-up interview on policymakers' perspectives on community engagement and cannabis policy. [Note: My team will conduct interviews on the topics represented in this survey with 40-50 participants].
21. If you checked any of the boxes above, please provide your email address so my team can meet your request(s). Your contact information will be kept separately from your survey responses; thus, your identity will not be linked to your answers.
    a. Email address:
